# Long noncoding RNA HIKER regulates erythropoiesis in Monge’s disease via CSNK2B

**DOI:** 10.1172/JCI165831

**Published:** 2023-06-01

**Authors:** Priti Azad, Dan Zhou, Hung-Chi Tu, Francisco C. Villafuerte, David Traver, Tariq M. Rana, Gabriel G. Haddad

**Affiliations:** 1 Division of Respiratory Medicine, Department of Pediatrics, and; 2 Department of Cell and Developmental Biology, UCSD, La Jolla, California, USA.; 3 Oxygen Transport Physiology Laboratory/Comparative Physiology, Faculty of Sciences and Philosophy, Cayetano Heredia University, Lima, Peru.; 4 Division of Genetics, Department of Pediatrics, Program in Immunology, Institute for Genomic Medicine, and; 5 Department of Neurosciences, UCSD, La Jolla, California, USA.; 6 Rady Children’s Hospital, San Diego, California, USA.

**Keywords:** Genetics, Hematology, Hypoxia, Noncoding RNAs

## Abstract

Excessive erythrocytosis (EE) is a major hallmark of patients suffering from chronic mountain sickness (CMS, also known as Monge’s disease) and is responsible for major morbidity and even mortality in early adulthood. We took advantage of unique populations, one living at high altitude (Peru) showing EE, with another population, at the same altitude and region, showing no evidence of EE (non-CMS). Through RNA-Seq, we identified and validated the function of a group of long noncoding RNAs (lncRNAs) that regulate erythropoiesis in Monge’s disease, but not in the non-CMS population. Among these lncRNAs is hypoxia induced kinase-mediated erythropoietic regulator (HIKER)/*LINC02228*, which we showed plays a critical role in erythropoiesis in CMS cells. Under hypoxia, HIKER modulated CSNK2B (the regulatory subunit of casein kinase 2). A downregulation of HIKER downregulated *CSNK2B*, remarkably reducing erythropoiesis; furthermore, an upregulation of *CSNK2B* on the background of HIKER downregulation rescued erythropoiesis defects. Pharmacologic inhibition of CSNK2B drastically reduced erythroid colonies, and knockdown of *CSNK2B* in zebrafish led to a defect in hemoglobinization. We conclude that HIKER regulates erythropoiesis in Monge’s disease and acts through at least one specific target, CSNK2B, a casein kinase.

## Introduction

Monge’s disease or chronic mountain sickness (CMS) is a clinical syndrome caused by chronic (years) exposure to high-altitude hypoxia, such as experienced by people living at Cerro de Pasco in the Andes (1–4). Excessive erythrocytosis (EE) (Hb ≥21 g/dL in men, Hb ≥19 g/dL in women) is the main feature of CMS, and this excessive pathobiological response to hypoxia has deleterious effects, since a high hematocrit/hemoglobin increases blood viscosity and reduces blood flow to hypoxia-sensitive organs (e.g., brain and heart), often resulting in myocardial infarction, stroke, and high mortality in young adults (1, 3–7). Remarkably, there are individuals who live at the same geographic location and altitude as the CMS subjects but are adapted and do not show any of the traits of the CMS individuals (henceforth called adapted or non-CMS). Investigating genetic and epigenetic mechanism(s) related to red blood cell differentiation, production, and cell death under hypoxic conditions in the CMS and non-CMS subjects would provide unique insights into the intricate regulation of erythropoiesis not only in these two groups of Andean highlander subjects, but possibly in normal human biological settings.

To understand the pathobiology of EE in CMS and the lack thereof in non-CMS subjects, we first performed whole-genome analysis of more than 100 subjects (CMS and non-CMS) to explore the idea that there are specific DNA-selected regions in the non-CMS population that have evolved over thousands of years to help non-CMS subjects adapt to the high-altitude environment. We then assessed the genes in these DNA-selected regions that could play an important role in Monge’s disease (8, 9). In order to assess the role of such genes, we first used PBMCs and induced pluripotent stem cell–derived (iPSC-derived) CD34^+^ cells from both groups and successfully replicated the hypoxia-induced EE phenotype in the CMS cell lines in vitro (6, 7). Discovering candidate genes and replicating the phenotype in vitro have allowed us to understand the role of such candidate genes in the phenotype. For example, we have studied the critical role of SENP1 in the CMS EE and the effect of this deSUMOylase on the activation of GATA1 (6). In the non-CMS group, we recently deciphered how ARID1B suppresses the erythropoietic response to hypoxia, involving chromatin modulation (5). These studies also demonstrated that the intricate regulation and dynamic changes of transcription in the erythroid lineage cells at specific stages are largely orchestrated by transcriptional factors (TFs), such as GATA1.

Since erythroid differentiation is highly regulated at the level of transcription (10, 11), we performed an RNA-Seq analysis of the erythroid cells (ex vivo using PBMC-derived native CD34^+^ cells) to study the transcriptional differences between the CMS and non-CMS subjects under normoxic and hypoxic conditions. We discovered differentially expressed changes in both coding and long noncoding RNAs (lncRNAs) and studied their role in erythropoiesis. We observed distinct profiles of lncRNAs in CMS and non-CMS cells in hypoxia and studied the role of the most upregulated lncRNA, HIKER/*LINC02228*, in the CMS cells. Knockdown (KD) of HIKER/*LINC02228* and its downstream target, the casein kinase 2 (CK2) β unit (CSNK2B), caused a severe reduction of EE response in CMS cells under hypoxic conditions. Consistent with these results, we observed that inhibition of CK2 activity resulted in a strong reduction of EE response in CMS cells. Based upon its role and specific mediating mechanism in regulating erythropoiesis in CMS cells, we have named this lncRNA HIKER — hypoxia induced kinase-mediated erythropoietic regulator. Our results show, for what we believe is the first time, that a lncRNA regulates erythropoiesis and that one of its targets, a casein kinase, plays an essential role in Monge’s disease.

## Results

### Differences in long noncoding expression among CMS and non-CMS subjects.

**Figure 1:**
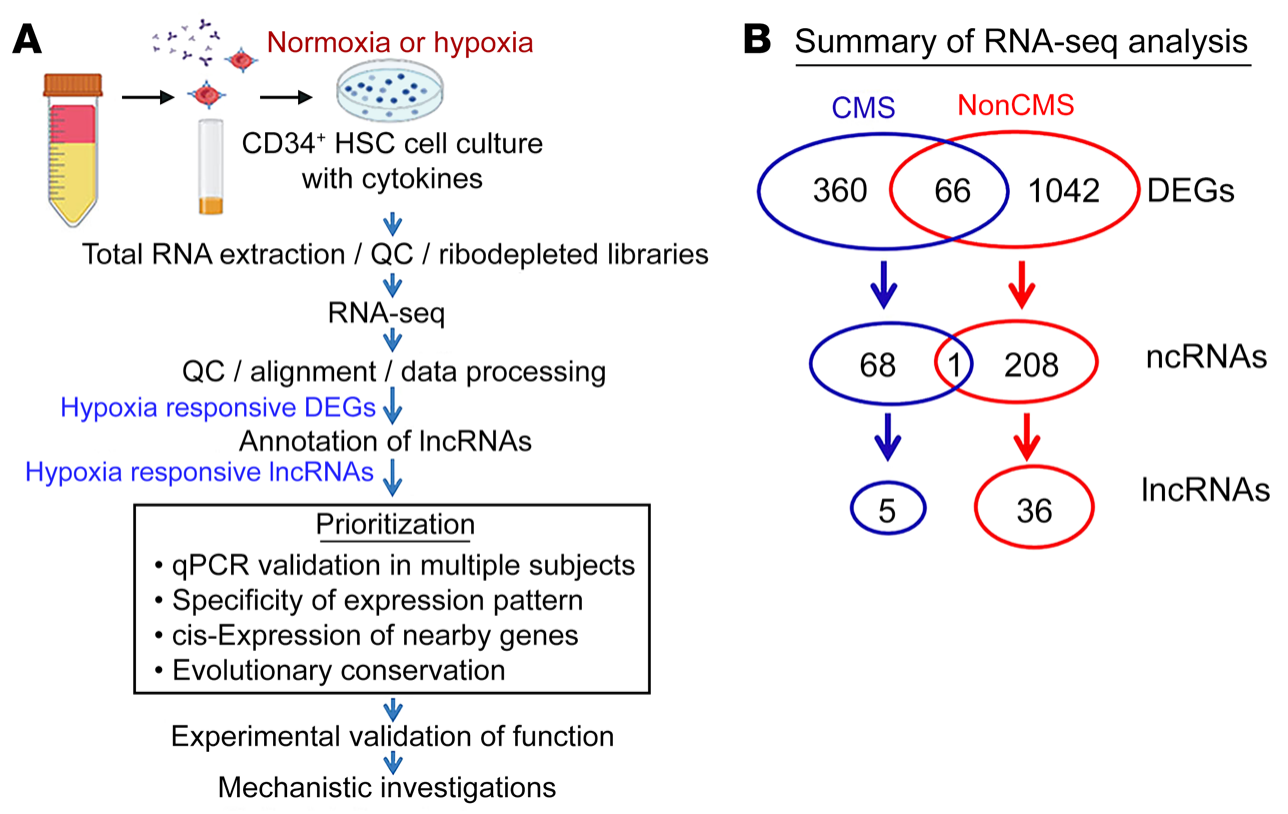
Schematic illustration of the experimental strategy and summary of RNA-Seq results. (**A**) CD34^+^ cells were isolated from blood (PBMCs) obtained from CMS or non-CMS subjects, pooled, and treated with hypoxia or room air (as control). Following the treatment, total RNA was isolated, and the quality was determined with TapeStation. Ribosome-depleted (ribodepleted) libraries were generated and sequenced. The candidate hypoxia-responding lncRNAs were identified and prioritized for further qRT-PCR–based evaluation and functional analyses. (**B**) Hypoxia treatment induced distinct transcriptional responses in CMS and non-CMS cells. A total of 426 or 1,702 hypoxia-induced DEGs were identified in the CMS and non-CMS cells, respectively, with little overlap. Further annotation revealed a distinct group of 5 lncRNAs in the CMS DEGs and 36 lncRNAs in the non-CMS DEGs, suggesting specific lncRNA-mediated hypoxia responses between CMS and non-CMS subjects (see also Supplemental Table 1).

**Figure 2:**
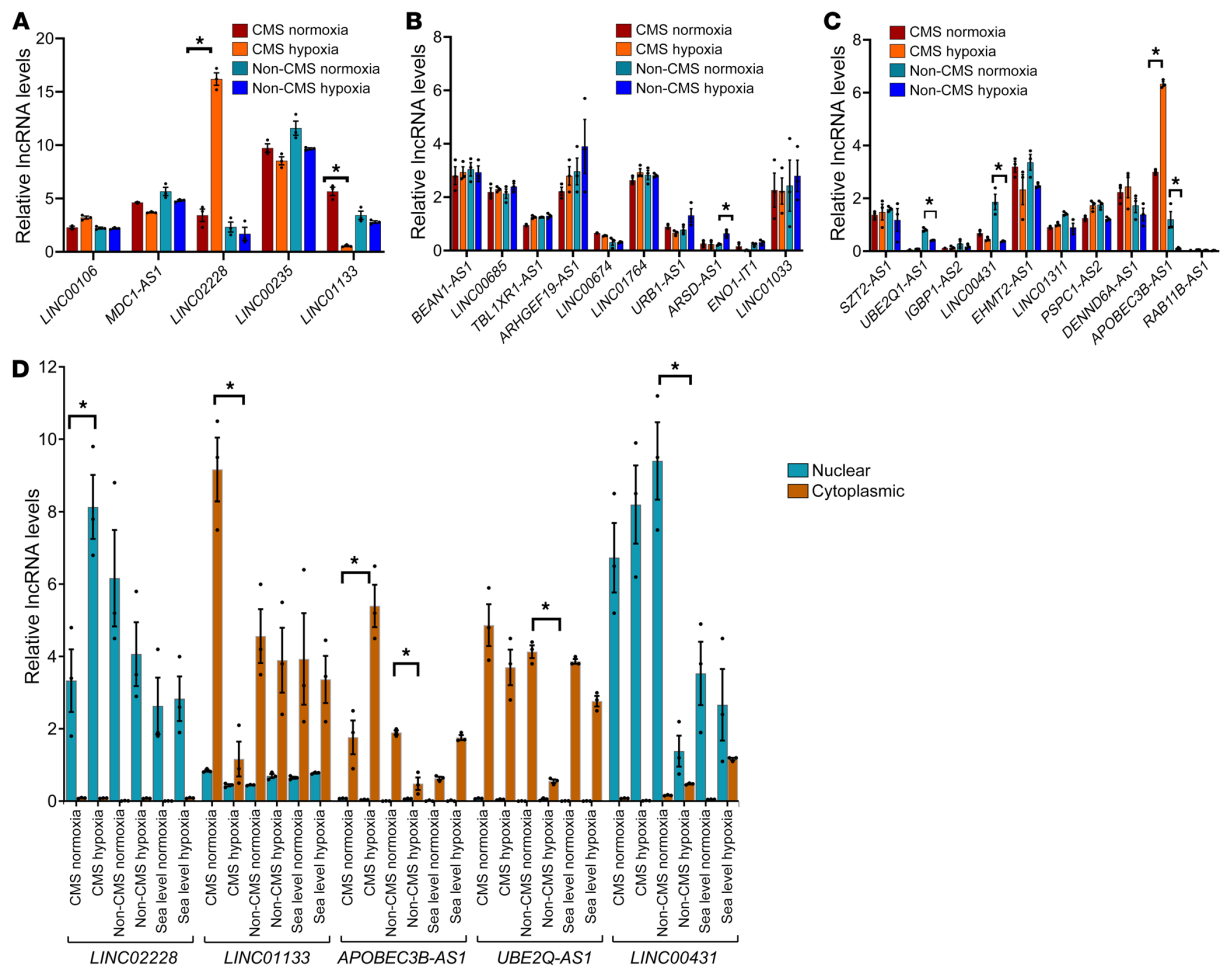
Validation of lncRNA expression changes in CMS and non-CMS cells under hypoxia and normoxia. (**A**) qRT-PCR validation of all 5 lncRNAs that were differentially altered (up- and downregulated) in the CMS cell group (Supplemental Table 1). iPSC-derived CD34^+^ cells after exposure to hypoxia and normoxia (for 3 days) were used for this assay. Expression levels were tested and validated in both CMS (*n* = 3) and non-CMS (*n* = 3) cells under hypoxia and normoxia. *P < 0.05, *t* test. HIKER/*LINC02228* was tremendously upregulated in the CMS cells under hypoxia. (**B**) qRT-PCR validation of top 10 upregulated lncRNAs in the non-CMS cell group (Supplemental Table 1). iPSC-derived CD34^+^ cells after exposure to hypoxia and normoxia (for 3 days) were used for this assay. Expression levels were tested and validated in both CMS (*n* = 3) and non-CMS (*n* = 3) cells under hypoxia and normoxia. *P < 0.05, *t* test. (**C**) qRT-PCR validation of top 10 downregulated lncRNAs in the non-CMS cell group (Supplemental Table 1). iPSC-derived CD34^+^ cells after exposure to hypoxia and normoxia (for 3 days) were used for this assay. Expression levels were tested and validated in both CMS (*n* = 3) and non-CMS (*n* = 3) cells under hypoxia and normoxia. *P < 0.05, *t* test. (**D**) Nuclear and cytoplasmic localization of lncRNAs. qRT-PCR results of confirmation for the expression changes for HIKER/*LINC02228*, *LINC00431* (nuclear) and *LINC01133*, and *APOBEC3B-AS1* and *UBE2Q1-AS1* (cytoplasmic) for CMS, non-CMS and sea-level erythroid cells under hypoxia and normoxia. iPSC-derived CD34^+^ cells after exposure to hypoxia and normoxia (for 3 days) were used for this assay. *P < 0.05, *t* test. *n* = 3 subjects for each group.

**Figure 3:**
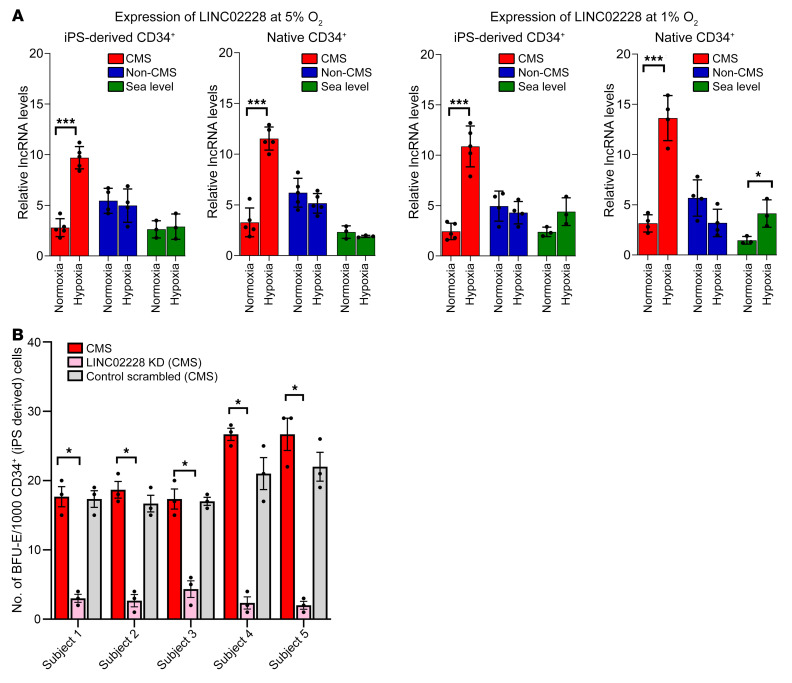
Critical role of HIKER/*LINC02228* in regulating hypoxia-induced erythropoietic response in CMS patients. (**A**) qRT-PCR results confirming the expression changes for HIKER/*LINC02228* for CMS, non-CMS, and sea-level subjects under normoxia and in response to hypoxia in iPSC-derived and PBMC-derived native CD34^+^ cells. Left panels show the expression of *LINC02228* at 5% O_2_, and right panels show the results at 1% O_2_. The figure shows the expression changes in iPSC-derived CD34^+^ cells as well as native CD34^+^ cells, as labeled in the figure. Native CD34^+^ cells: *n* = 5 (CMS and non-CMS cells); *n* = 3 (sea-level subjects). iPSC-derived CD34^+^ cells: *n* = 5 (CMS and non-CMS cells); *n* = 3 (sea-level subjects). *P < 0.05; ***P < 0.01, *t* tests for comparison with hypoxia and normoxia values for each sample. (**B**) Functional analysis of HIKER/*LINC02228* in iPSC-derived CD34^+^ cells using methylcellulose colony assay. Panel shows significant reduction of BFU-E under hypoxia with KD of each lncRNA in CMS in each subject. *n* = 5 subjects. Each subject was tested at least 3 times. *P < 0.01. One-way ANOVA was performed in multiple comparisons, followed by Tukey’s tests.

We exposed PBMC-derived native CD34^+^ cells that were isolated from CMS (*n* = 4) and non-CMS (*n* = 2) subjects to either 5% O_2_, a hypoxia level that induces significant EE in CMS (5), or normoxia (as controls) (Figure 1A). Since we intended to start with a screening experiment, we pooled samples for each group and performed an RNA-Seq on the pooled samples to determine the transcriptomic response of the CMS and non-CMS cells to hypoxia. We identified a total of 360 differentially expressed genes (DEGs) in the CMS cells and 1,042 DEGs in the non-CMS cells (>2-fold), including both coding and lncRNAs. Among these DEGs, we identified 5 differentially expressed lncRNAs in CMS and 36 differentially expressed lncRNAs in non-CMS with no overlap (Figure 1B and Supplemental Table 1; supplemental material available online with this article; https://doi.org/10.1172/JCI165831DS1). Such distinct differences in the transcriptional response in lncRNAs between CMS and non-CMS suggested that specific lncRNA mechanisms are involved in stimulating or inhibiting hypoxia-induced erythropoiesis. To verify the expression of a subset (top up- and downregulated based on fold change; Supplemental Table 1) of the candidate lncRNAs, we used real-time PCR in iPSC-derived CD34^+^ cells that were generated from CMS (*n* = 3) and non-CMS (*n* = 3) subjects (Figure 2, A–C). Significantly altered lncRNAs under hypoxia included HIKER/*LINC02228*, *LINC01133*, *ARSD-AS1*, *UBE2Q1-AS1*, *RAB11-B-AS1*, *LINC00431*, and *APOBEC3B-AS1* (Figure 2, A–D). In addition, the hypoxia-induced upregulation of HIKER/*LINC02228* was confirmed in another set of iPSC-derived and PBMC-derived native CD34^+^ cells obtained from CMS (*n* = 5) or non-CMS (*n* = 5) subjects (Figure 3A) at 5% O_2_. We further tested HIKER/*LINC02228* expression levels in iPSC-derived and PBMC native CD34^+^ levels at 1% O_2_ and found a similar response in all the samples (Figure 3A). Since lncRNAs can be predominantly either in the cytosol or in the nucleus, we verified the cellular distribution of the top 5 significantly changed lncRNAs and found that HIKER/*LINC02228* and *LINC00431* are predominantly located in the nucleus whereas *LINC01133*, *UBE2Q1-AS1,* and *APOBEC3B-AS1* are mostly cytoplasmic in location (Figure 2D). Since we focused in the current study on transcriptional regulation, we applied nuclear-specific approaches to studying the functional role of the nuclear lncRNAs (i.e., HIKER/*LINC02228* and *LINC00431*) in regulating erythropoiesis.

### HIKER/LINC02228 regulates erythropoiesis in CMS subjects.

In order to assess the functional role of the candidate nuclear lncRNAs, we selected lncRNAs that were upregulated in CMS cells (i.e., HIKER/*LINC02228*) (Figure 2A, Figure 3A, and Supplemental Figure 2) and downregulated in non-CMS cells (i.e., *LINC00431*) (Supplemental Figure 2). By testing the function (by colony-forming assay) of these 2 lncRNAs in CMS cells, we sought to ascertain the functionality as well as specificity of each candidate lncRNA. Using the efficient available KD strategy for nuclear lncRNAs (12), we downregulated the 2 lncRNAs using antisense oligonucleotide (ASO) (as detailed in Methods). The downregulation (>80%, Supplemental Figure 1) of HIKER/*LINC02228* in the CMS cells led to a significant reduction of burst-forming unit–erythroid (BFU-E) colonies (*P* < 0.0001) (Figure 3B), but only a modest suppression (*P* = 0.043) (Supplemental Figure 2) by *LINC00431* with no statistical significance against the scrambled control (*P* > 0.05) (Supplemental Figure 2). These results demonstrate a critical role of HIKER/*LINC02228* in regulating erythroid progenitors (BFU-E) in CMS cells under hypoxia. The strong inhibition of BFU-E progenitors with KD of HIKER/*LINC02228*, but not with KD of *LINC00431*, also strongly suggests a specific role of HIKER/*LINC02228* in regulating EE in CMS subjects.

### CSNK2B is a critical mediator of HIKER/LINC02228 for driving erythropoiesis under hypoxia.

**Figure 4:**
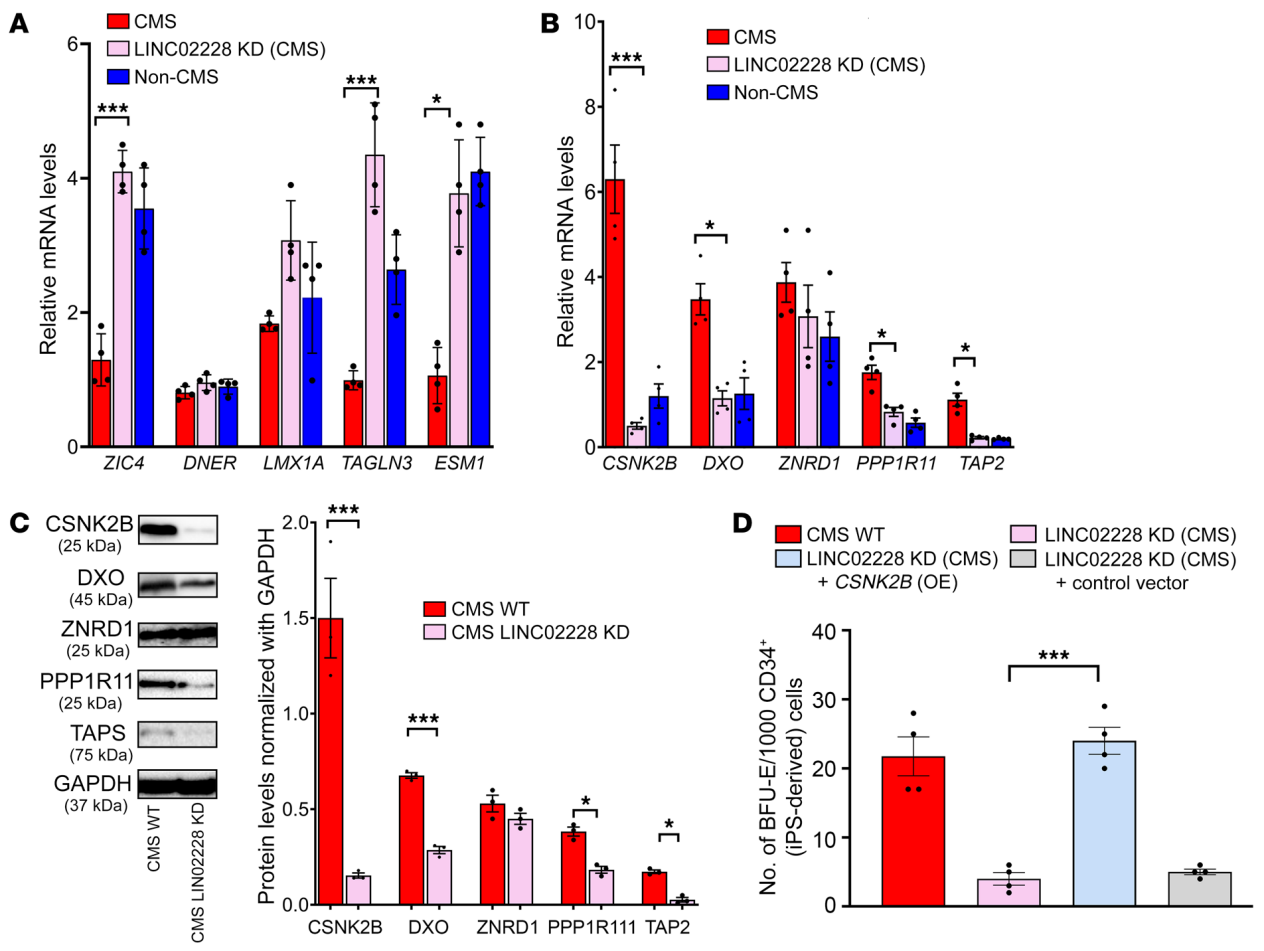
HIKER regulates erythropoietic response via CSNK2B. (**A**) qRT-PCR results confirming expression changes for RNA-Seq analysis of the KD of HIKER/*LINC02228* versus controls. Top 5 upregulated genes are shown. qPCR was performed on iPSC-derived CD34^+^ cells. *n* = 4 subjects per group. *P < 0.05; ***P < 0.001. *t* tests were performed to compare expression levels of CMS (WT) with CMS (KD of HIKER) for each gene. (**B**) qRT-PCR results confirming the expression changes for RNA-Seq analysis of the KD of HIKER/*LINC02228* versus controls. Top 5 downregulated genes are shown. qPCR was performed on iPSC-derived CD34^+^ cells. *n* = 4 subjects per group. *P < 0.05; ***P < 0.001. *t* tests were performed to compare the expression levels of CMS (WT) with CMS (KD of *LINC02228*/HIKER) for each gene. (**C**) Western blot confirmation of the top 5 downregulated candidates, CSNK2B, DXO, ZNRD1, PP1R11, and TAP2. Week 1 EBs (iPSC derived) were used in this assay as described in Methods. Left: representative image for each protein candidate. Right: summary of densitometric analysis of each protein with *n* = 3 for each group. *P < 0.05; ***P < 0.001; *t* test was performed to compare the protein levels with CMS (WT) and CMS (KD of LINC02228/HIKER). (**D**) Functional analysis of HIKER/LINC02228 as well as CSNK2B-OE-LINC02228-KD in iPSC-derived CD34^+^ cells using methylcellulose colony assay. With the OE of CSNK2B gene in the background of HIKER/LINC02228 KD, mean number of BFU-E colonies/CD34^+^ is increased, suggesting a critical function of this gene in the mechanism of action of HIKER/LINC02228. *n* = 4 subjects per group. ***P < 0.001. One-way ANOVA was performed in multiple comparisons followed by Tukey’s tests.

To determine potential downstream factors mediating the function of HIKER/*LINC02228* in erythropoiesis, we next identified DEGs in the CMS cells following the KD of HIKER/*LINC02228* or *LINC00431*. Compared with controls, we identified a total of 363 DEGs with HIKER/LIN02228 KD and a total of 361 DEGs with LIN00431 KD. Since HIKER/LINC02228 KD specifically decreased hypoxia-induced BFU-E colonies, but LINC00431 KD had no significant effect (Figure 3 and Supplemental Figure 2), we used the list of LINC00431 KD DEGs as an additional filtering strategy for identifying the DEG candidates that were specifically altered by HIKER/LINC02228 KD, as these would be more likely to be functional mediators of *LINC02228* in excessive erythropoiesis. To do so, we compared the list of DEGs following HIKER/LINC02228 KD or LINC00431 KD and removed 238 DEGs that were common in both lists. This filtering process generated a list of 125 candidate DEGs that were specifically altered by HIKER/LINC02228 KD (Supplemental Table 2), which we focused on in the follow-up studies. The top upregulated candidates (i.e., *ZIC4*, *DNER*, *LMX1A TAGLN3*, and *ESM1*) were then verified using quantitative PCR (qPCR), and the most downregulated candidates (i.e., *CSNK2B*, *DXO*, *ZNRD1*, *PPP1R11* and *TAP2*) were verified with both qPCR and Western blot analysis (Figure 4, A–C, Supplemental Figure 4, and Table 1). Through filtering and experimental validation processes, we confirmed that CSNK2B was a promising candidate with the most significant (*P* < 0.01) alterations by both qPCR and Western blotting. In order to functionally assess (through colony-forming assay) whether CSNK2B is a critical mediator of HIKER/*LINC02228*, we performed a rescue experiment (Figure 4D) in which we knocked down HIKER/LINC02228 in CMS cells, and on such a background, we overexpressed (OE) CSNK2B. Indeed, CSNK2B OE completely rescued the effect of HIKER/LINC02228 KD, demonstrating that CSNK2B is a critical downstream effector mediating the function of HIKER/LINC02228 (Figure 4D).

### CSNK2B is an erythropoietic regulator in CMS and non-CMS cells.

**Figure 5:**
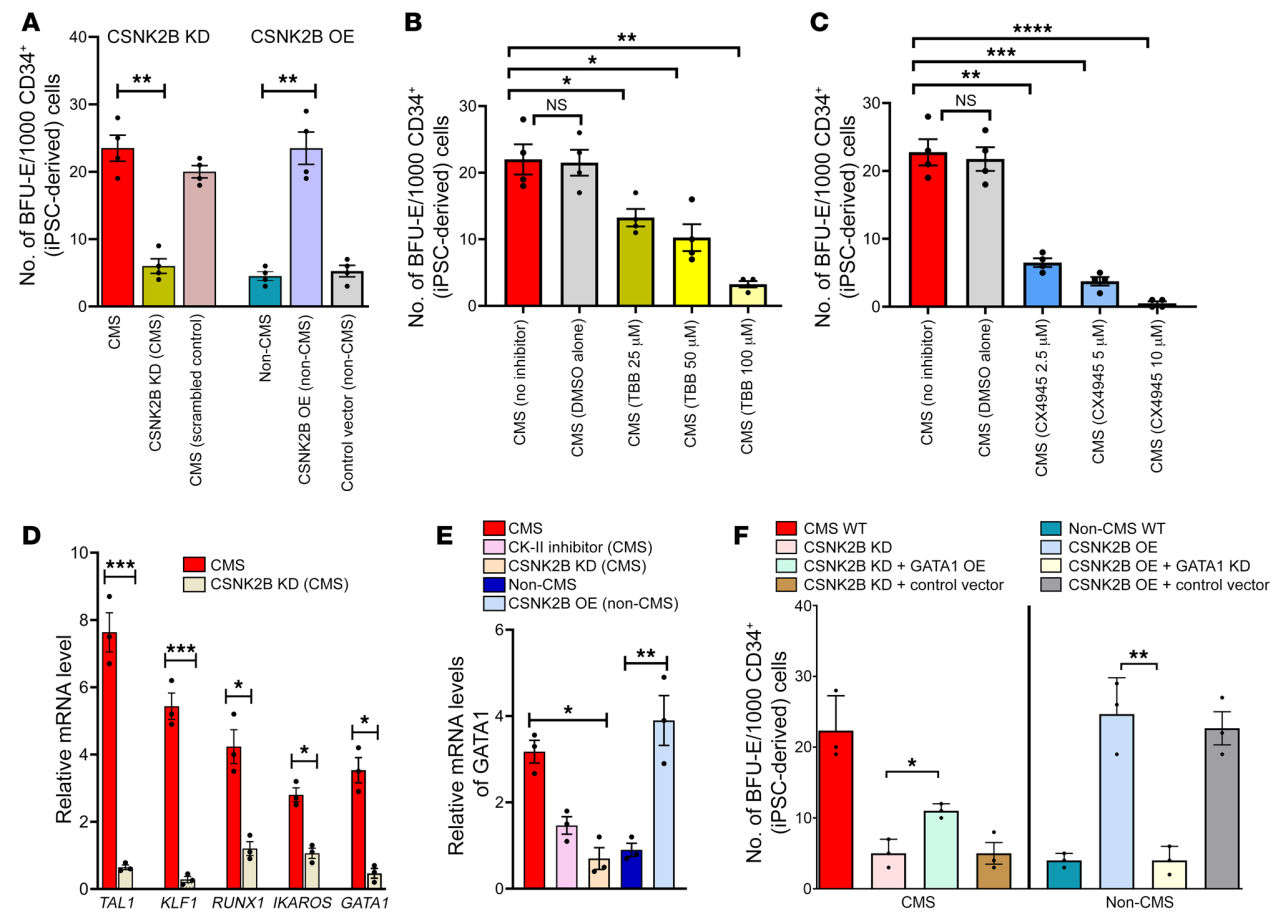
CSNK2B plays an important role in regulating erythropoiesis at high altitude partially through GATA1. (**A**) CSNK2B KD in CMS decreases BFU-E, and CSNK2B OE in non-CMS increases BFU-E, suggesting its critical role in regulating erythropoiesis. **P < 0.001. (**B**) Effect of CK2 inhibitor on CMS cells. TBB decreases BFU-E colonies in CMS cells in a dose-dependent manner. *P < 0.05; **P < 0.001. (**C**) Effect of CK2 inhibitor on CMS cells. CX4945 decreases BFU-E colonies more drastically in the CMS cells in a dose-response manner. **P < 0.01; ***P < 0.001; ****P < 0.0001. (**D**) CSNK2B KD results in major expression changes of critical TFs. qPCR results confirm decreased expression of *TAL1*, *KLF1*, *RUNX1*, *IKAROS*, and *GATA1*. *P < 0.05; ***P < 0.001. (**E**) *GATA1* expression levels were altered significantly by modulation of CSNK2B levels in CMS and non-CMS cells under hypoxia. Graph shows *GATA1* expression as measured by qPCR in (a) CMS cells, (b) CMS cells with CSNK2B KD, (c) CMS cells treated with CK2 inhibitor, (d) non-CMS cells, and (e) non-CMS cells with CSNK2B-OE. *P < 0.05; **P < 0.01. (**F**) CSNK2B regulates erythropoietic response through GATA1. Graph shows the effect of CSNK2B and GATA1 modulation on colony-forming potential of CMS and non-CMS cells. GATA1 OE partially rescues the erythropoietic suppression caused by CSNK2B in CMS. Further, KD of GATA1 in non-CMS results in loss of excessive erythropoiesis caused by OE of CSNK2B. *P < 0.05; **P < 0.001. For all the experiments (colony-forming assays as well as qPCR), iPSC-derived CD34^+^ cells were used. *n* = 3 per group. For **A**–**F**, 1-way ANOVA was performed, followed by multiple comparisons by Tukey’s test. For **D**, *t* tests were performed for each comparison.

We further evaluated the role of CSNK2B in erythropoiesis using our in vitro erythroid platform. On the one hand, when we downregulated *CSNK2B* expression in CMS cells, there was a remarkable decrease in erythropoiesis in response to hypoxia. On the other hand, *CSNK2B* OE in the non-CMS cells resulted in an excessive erythropoietic response to hypoxia, which phenocopied the CMS cells (Figure 5A). In order to test our hypothesis that the role of CSNK2B in EE is achieved through its regulation of CK2 activity, we used specific inhibitors (TBB and CX-4945) of the CK2 and studied their effect on erythroid colony production. Consistently with the KD results, we observed significant changes in the colony numbers in a dose-dependent manner with the inhibitors (Figure 5, B and C) (*P* < 0.0001, multiple comparisons by Tukey’s test between control and inhibitor at various dosages). Collectively, RNAi and inhibitor results confirm an important role of CK2 in regulating the erythropoietic response of CMS and non-CMS cells under hypoxia.

### CSNK2B mediates the high-altitude erythropoietic response in part through GATA1.

In order to determine how CSNK2B regulates erythropoiesis, we performed RNA-Seq of CNSK2B KD (CMS) versus control (CMS, no KD). Remarkably, we found that several critical TFs (i.e., TAL1, KLF1, and GATA1) as well as the erythropoietin receptor (EPOR, a target of HIF1A) were downregulated (>2-fold) by CSNK2B KD in CMS cells (Figure 5D). Since (a) GATA1 is a major erythroid-specific TF and can regulate the expression of other erythroid target genes such as TAL1 and KLF1 (13–17) and (b) we have previously shown that GATA1 plays a critical role in regulating erythropoiesis in CMS cells (5, 9), we further performed experiments to investigate whether CSNK2B functions via GATA1. First, we measured the expression levels of *GATA1* in CMS cells and non-CMS cells after CSNK2B KD. In CMS cells, the KD of CSNK2B as well as pharmacologic inhibition of CK2 resulted in downregulation (about 2-fold, *P* < 0.05) of *GATA1* levels (Figure 5E). In the non-CMS cells, however, OE of CSNK2B led to upregulation (about 3-fold, *P* < 0.001) of *GATA1* levels (Figure 5E). Second, in order to show that there is a functional interaction between CSNK2B and GATA1, we used double mutants of CSNK2B and GATA1 and analyzed their effect on colony formation. We observed that GATA1OE was able to partially rescue the erythropoietic suppression caused by CSNK2B in CMS cells (Figure 5F). In non-CMS cells, the KD of GATA1 led to a large (>5-fold) decrease in the excessive erythropoietic response (BFU-E colonies) caused by CSNK2B OE in these cells (Figure 5F). The control vectors by themselves did not affect the phenotypes, implying an important role of GATA1 in this experiment (Figure 5F). These results confirm a partial role of GATA1 as a downstream mediator of CSNK2B in regulating erythropoiesis in CMS and non-CMS cells under hypoxia.

### CSNK2B KD induces severe hemoglobinization defect in zebrafish embryos.

**Figure 6:**
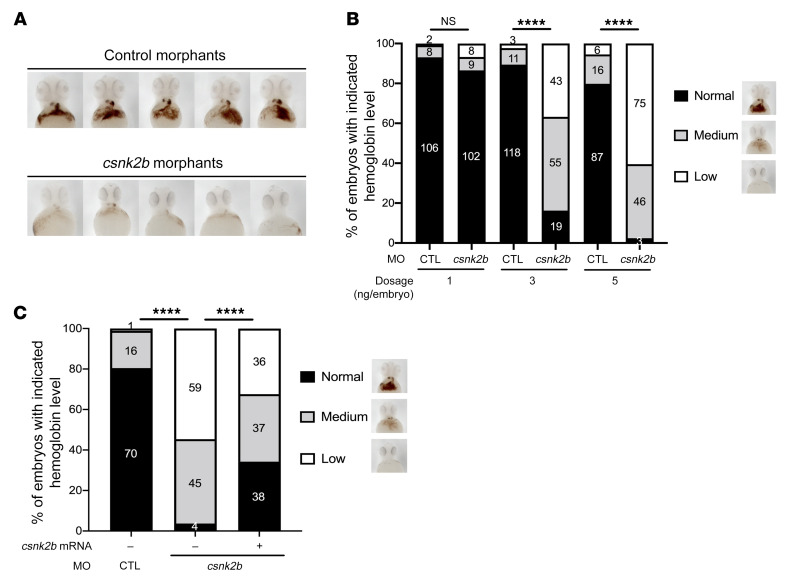
CSNK2B plays a critical role in regulating erythropoiesis in vivo. (**A**) Csnk2b is required for hemoglobinization of zebrafish erythrocytes. Representative images of hemoglobin signal in control and csnk2b morphants stained with *o*-dianisidine at 2 dpf. Images shown are ventral views with heads to the top. (**B**) Statistical analyses showing dose-dependent loss of hemoglobin in embryos injected with 1, 3, or 5 ng of control (CTL) or csnk2b morpholino. (**C**) Statistical analysis of hemoglobin phenotypes in control and csnk2b morphants with or without rescue of *csnk2b* mRNA. Representative images of hemoglobin classification criterion are shown on the right side of the graph. Data collected from 3 independent experiments, with corresponding embryo numbers displayed on the columns. ****P < 0.0001. For **B** and **C**, χ^2^ tests were performed to examine the statistical difference of the phenotype distribution between groups.

Since the CSNK2B protein sequence is 99% conserved between humans and zebrafish (Supplemental Figure 3), we assessed the role of CSNK2B in erythropoiesis in vivo during zebrafish development. We knocked down the expression of *csnk2b* in zebrafish embryos using a morpholino antisense oligonucleotide (MO) that blocks the translation of *csnk2b*. Compared with controls, *csnk2b*-KD embryos displayed a remarkable decrease in hemoglobin at 3 ng and 5 ng MO dosage, 2 days post fertilization (dpf) (Figure 6, A and B). A few of the morphants displayed normal iron incorporation at these dosages (Figure 6, A and B). On the other hand, more than 97% of morphants showed moderate or low iron staining, indicating a key role of CSNK2B in the maturation of the RBC lineage. Moreover, we found a dose-dependent increase in phenotype severity (Figure 6B), with a minimal impact on hemoglobin levels at 1 ng dose, but increasing severity in hemoglobin levels at 3 ng and 5 ng. Furthermore, hemoglobin levels in *csnk2b* morphants were rescued via coinjection of *csnk2b* mRNA (Figure 6C). Taken together, these findings indicate that CSNK2B plays a key role in erythropoiesis.

Our results show distinct expressional changes in lncRNAs under hypoxia in CMS and non-CMS cells. We also prove, for what we believe is the first time, that the lncRNA HIKER/*LINC02228* regulates the excessive erythropoiesis of Monge’s disease (Figure 3) and that its action is mediated through CSNK2B, a casein kinase. Furthermore, in vivo KD of CSNK2B in zebrafish results in severe reduction in hemoglobin, further proving its vital role in erythropoiesis.

## Discussion

The noncoding portion of the genome is actively transcribed, and thousands of regulatory short and lncRNAs (e.g., snoRNAs or lncRNAs) are generated to regulate gene networks under physiological or pathological conditions (18–24). Indeed, accumulating evidence has demonstrated that lncRNAs play significant roles in human pathobiology, including immunological, neurological, cardiovascular, and respiratory diseases and cancer as well as developmental disorders (18, 20–25). In the current study, we demonstrate an important role of lncRNAs in the excessive erythropoiesis of Monge’s disease. We identified a group of hypoxia-responding lncRNAs in the CD34^+^ cells that include HIKER/*LINC02228* and *LINC01133* in the CMS as well as *ARSD-AS1*, *UBE2Q1-AS1*, *LINC00431*, and *APOBEC3B-AS1* in the non-CMS subjects (Figure 2 and Supplemental Table 1). One important discovery was that the differentially expressed lncRNAs were unique between CMS and non-CMS with no overlaps, indicating that hypoxia (high altitude) evoked distinct lncRNA mechanisms that regulate the development and progression of EE in CMS or the protection or resistance to EE in non-CMS cells. This notion was clearly demonstrated by HIKER/*LINC02228*, as it exhibited a specific response to hypoxia in CMS cells and not in the non-CMS (or the sea-level control cells) (Figure 2), again indicating that the mechanisms underlying CMS and non-CMS are different, as in previous work from our laboratory (5, 6).

Previous studies have shown that lncRNAs regulate gene expression at both the transcriptional and posttranscriptional levels through a variety of mechanisms (25–27) and their subcellular distribution informs their mechanism of action (25–27). Through a rigorous experimental and bioinformatic approach combining RNA-Seq–based screen, data filtering/prioritization, experimental validation, and rescue assays, we demonstrate that CSNK2B is a critical downstream mediator of HIKER/*LINC02228* in excessive erythropoiesis in CMS patients. Furthermore, the role of CSNK2B in regulating erythropoietic response was functionally evaluated in both CMS and non-CMS cells (Figure 5) and in vivo in zebrafish (Figure 6, A–C). Interestingly, a recent study, published in an abstract form, also showed that Csnk2b is a critical regulator of erythropoiesis in mice (28). In this mouse model with a hematopoietic-specific conditional KO of *Csnk2b* (i.e., Vav1-CRE × Csnk2β^fl/fl^ mice), Piazza’s and colleagues found that *Csnk2b* deficiency was lethal in utero and that fetuses displayed a severe anemic phenotype, suggesting that loss of *Csnk2b* altered erythroid development and led to defects of red cell viability (28). Our results in this work (humans and zebrafish) as well as Piazza’s work in mice hence demonstrate that CSNK2B is evolutionarily conserved. Since (a) we show in this current study that CSNK2B acts as a target of HIKER, playing an important role in Monge’s disease (29–31) and (b) application of selective CK2 inhibitors (32–34) decreased BFU-E colonies in a dose-dependent manner (*P* < 0.01), inhibition of CK2 activity is a potentially effective therapeutic strategy for treating excessive erythropoiesis in CMS subjects at high altitude.

lncRNAs can play a role in early as well as late erythroid stages depending on their interaction with stage-specific TFs (35–38). In this work, we have focused mostly on the role of HIKER in early erythroid progenitors, specifically BFU-E. It is possible that HIKER can affect multiple erythroid stages and other lncRNAs can affect other stages as well, and therefore, further studies are required for understanding all stage-specific effects of HIKER or other lncRNAs in erythropoiesis in Monge’s disease.

Our previous studies have shown a critical role for GATA1 in regulating the excessive erythropoiesis in Monge’s disease. This role of GATA1 is mediated through SENP1 deSUMOylase activity (6) or changes in chromatin accessibility and its effect on *GATA1* expression (5). In our current study, we observed that CSNK2B also functions in part through GATA1 mediation. Although GATA1 rescued the erythropoietic suppression induced by CSNK2B KD in CMS cells, it was a partial rescue. This could be due to the fact that other mediators (such as TAL1 and KLF1) (Figure 5) are responsible for the rescue as well. In the non-CMS cells, the effect of colony formation in GATA1 KD and on the background of CSNK2B OE was large, suggesting that GATA1 is a downstream erythropoietic mediator of CSNK2B in non-CMS cells. Furthermore, it has been shown that CK2 inhibition was associated with decreased HIF-1 activity (39–45). Indeed, we detected decreased expression of *EPOR* (a downstream target of HIF-1) (46–48) following CSNK2B KD, suggesting that, in addition to GATA1, HIF signaling might be another potential mechanism underlying the role of CSNK2B in excessive erythropoiesis in Monge’s disease.

It is interesting to speculate about human adaptation to high altitudes, especially that some populations have lived at high altitudes for thousands of years. We have previously shown, for example, that ARID1B (5) curbs excessive erythropoiesis in the Andean population, but it is possible, as other authors have shown, that EPAS1 loss-of-function variants curb EE in the Tibetan population (49, 50). Interestingly, these variants were not identified in the Andean highlanders as a major adaptation mechanism. This is potentially due to founder effects, suggesting the existence of unique adaptation mechanisms in various highlander populations.

In summary, our study identified a group of lncRNAs and delineated the critical role of HIKER/*LINC02228* in the EE of Monge’s disease. Furthermore, we discovered that CSNK2B has an evolutionarily conserved role in erythropoiesis, but it is through CK2 activity that HIKER/*LINC02228* functions in humans. We believe that HIKER/*LINC02228* and CSNK2B/CK2 are potential novel therapeutic targets for the treatment of EE in CMS. As with previous studies of extreme phenotypes (51), the CSNK2B-mediated lncRNA HIKER/*LINC02228* function underlying the pathobiology of CMS at high altitude may pave the way for understanding erythropoiesis in other related diseases at sea level.

## Methods

### Patient samples.

All subjects used in this study (CMS and non-CMS) were adult males, lifelong residents of Cerro de Pasco, Peru, and living at an elevation of approximately 4,338 m. CMS patients fulfilled the diagnostic criteria for CMS, or Monge’s disease, based on hematocrit, O_2_ saturation, and CMS score, as described in detail in our previous studies (6, 52). Sea-level individuals used in this study are individuals who have permanently resided at sea level and are within the age group of CMS and non-CMS subjects.

### Native CD34^+^-derived erythroid cells.

Blood samples for PBMC isolation were obtained in sodium heparin–coated tubes. PBMCs were isolated using Histopaque 1077 (Sigma-Aldrich, 10771) by gradient centrifugation. The Dynabeads CD34^+^ Isolation Kit (Invitrogen, 11301D) was used to purify the CD34^+^ fraction. CD34^+^ cells were expanded for a week (days 0–7) in StemSpan medium (STEMCELL Technologies, 09600)containing hydrocortisone (MilliporeSigma, H6909), 50 ng/mL SCF (Peprotech, 300-07), 50 ng/mL FLT3L (Peprotech, 300-19), 10 ng/mL IL-3 (Peprotech, 200-03), 1 ng/mL BMP4 (Peprotech, 120-05), 40 ng/mL IL-11 (Peprotech, 200-11), and 2 U/mL EPO (Amgen, 55513014810). After expansion, cells were further differentiated using the protocol from Giarratana et al. (53). Briefly, cells were then cultured in erythroid differentiation medium (EDM), which includes IMDM supplemented with stabilized glutamine (MilliporeSigma, FG0465), 330 μg/mL holo-human transferrin (MilliporeSigma, T0665), 10 μg/mL recombinant human insulin (MilliporeSigma, I9278), 2 IU/mL heparin, and 5% plasma (Innovative Research, IPLAWBCPD).

### iPSC-derived erythroid cells.

The iPSC lines from CMS, non-CMS, and sea-level subjects have been generated and well characterized by us (5, 36). The iPSCs were thoroughly assessed using various methods, including DNA fingerprinting, high-resolution karyotyping, and alkaline phosphatase staining, as well as the expression of multilineage differentiation markers, as described in our previous publications (6, 52). We generated the erythroid cultures from iPSCs, following our previously established in vitro platform, based on the protocol of Douay’s group (54). We have previously studied in detail the characteristics of these generated erythroid cells of CMS and non-CMS subjects, including CD markers, maturation, and hemoglobin (6, 54). Briefly, we started the erythroid cultures with approximately 10^7^ to 10^8^ cells of human iPSC cell lines in all subjects. Human iPSCs were differentiated from erythroid cells by formation of embryoid bodies (EBs) for 27 days in a liquid culture medium with the base medium IMDM (MilliporeSigma, FG0465) along with 450 μg/mL holo human transferrin (MilliporeSigma, T0665), 10 μg/mL recombinant human insulin (MilliporeSigma, I9278), 2 IU/mL heparin (NDC 63739-920-25 purchased from McKesson), and 5% human plasma (Innovative Research, IPLAWBCPD) in the presence of 100 ng/mL SCF (Peprotech, 300-07), 100 ng/mL TPO (Peprotech, 300-18), 100 ng/mL FLT3 ligand (Peprotech, 300-19), 10 ng/mL rhu bone morphogenetic protein 4 (BMP4) (Peprotech, 120-05), 5 ng/mL rhu VEGF (Peprotech, 100-20), 5 ng/mL IL-3 (Peprotech, 200-03), 5 ng/mL IL-6 (PeproTech, 200-06), and 3 U/mL Epo (Amgen, 55513014810, purchased from McKesson). This was followed by terminal differentiation as single cells with base medium IMDM (Millipore Sigma, FG0465) along with 5% human plasma (Innovative Research, IPLAWBCPD), 2 IU/mL heparin (McKesson, NDC 63739-920-25), 100 ng/mL SCF (Peprotech, 300-07), 5 ng/mL IL-3 (Peprotech, 200-03), and 3 IU/mL EPO (Amgen, 55513014810).

### RNA-Seq and data analysis.

Native CD34^+^ cells were isolated from PBMCs as described above to determine differentially expressed lncRNAs. To do so, RNA was isolated from the erythroid cells after 3 days of exposure to hypoxia or normoxia in CMS (*n* = 4) and non-CMS (*n* = 2). RNA was isolated using the Zymo RNA Kit (Zymo, R1050) per the manufacturer’s instructions. The quality of RNA was assessed using TapeStation (Agilent). Ribosome depletion–prepared CMS or non-CMS samples were balanced pooled, and the sequencing libraries were generated by using the TruSeq Stranded Total RNA with RiboZero Gold Library Preparation Kit (Illumina, RS-122-2301). The ribosome-depleted prepared libraries were sequenced using the HiSeq 2500 System in Rapid Run mode (Illumina). A total number of approximately 50 million reads per library were obtained. The resulting reads were mapped using the RUM alignment package with default setting to the human reference hg38. The aligned reads were then processed with htseq-count to obtain the number of reads mapped to genes (Illumina’s iGenome GTF annotation for hg38). Quality control (QC) processes were performed prior to and after alignment to ensure high quality of final results. This included GC content, the presence of adaptors, FastQC (http://www.bioinformatics.babraham.ac.uk/projects/fastqc/) for sequence quality, overrepresented k-mers, and duplicated reads, and Picard (http://broadinstitute.github.io/picard/)/RseQC for mapping quality. Differentially expressed transcripts were determined by EBSeq (55). LNCipedia (https://lncipedia.org/) and GENCODE (https://www.gencodegenes.org/) were used for lncRNA annotation (19, 56–65).

To determine DEGs following HIKER/LINC02228-KD or LINC00431-KD, total RNA was isolated from the CMS iPSC-derived CD34^+^ with or without a KD of HIKER/LINC02228 or LINC00431 using the Zymo RNA Kit (Zymo, R1050), and the RNA-Seq libraries were generated using the Illumina TruSeq Stranded Total RNA Kit (Illumina, catalog RS-122-2301) per the manufacturer’s instructions. A total of more than 40 million reads per library were obtained following sequencing with the HiSeq 2500 System. After QC, the resulting reads were mapped using the RUM alignment package with default setting to the human reference hg38. Differentially expressed transcripts were determined by DESeq2 (66).

### Cellular fractionation and qPCR analysis of differentially expressed lncRNAs.

Briefly, total nuclear and cytoplasmic extracts were isolated from erythroid cultures (iPSC-derived CD34^+^ cells isolated from EBs as described in detail above) using Active Motif (catalog 40010) according to the manufacturer’s instructions. qPCR for HIKER/LINC02228, LINC01133, APOBEC3B-AS1, UBE2Q-AS1, and LINC00431 were used to assess the purity of the fractions. Primers are listed in Supplemental Table 3.

### KD of nuclear lncRNA HIKER/LINC02228 and LINC00431 expression using QIAGEN LNA gapmers ASO.

Locked nucleic acids (LNAs) targeting HIKER/LINC02228 and LINC00431 were designed and synthesized by Exiqon. Detailed sequences are listed in Supplemental Table 3. The most efficient ASO for each LNA was initially tested in the pilot experiment with and without transfection reagent (Lipofectamine 3000, Life Technologies, L3000-008) in a dose-response experiment at a concentration of 10 nM, 25 nM, 50 nM, and 100 nM. The uptake and the effect of ASO were monitored by qPCR at various stages (iPSC stage and CD34^+^ cells isolated from EBs). For both lncRNAs, the optimal delivery for all the stages was at the 50 nM concentration without the transfection reagent.

### Isolation of CD34^+^ cells from iPSC-derived EBs.

CD34^+^ cells were isolated from iPSC-derived EBs as follows. After 7 days of differentiation, EBs were harvested by spinning at 400*g* for 10 minutes. After centrifugation, EBs were dissociated into single cells using Accutase treatment for 10 minutes and then filtered through a 60 μm cell strainer (Falcon). CD34^+^ cells were isolated from this cell suspension using EasySep Human CD34 Positive Selection Kit II (STEMCELL Technologies, 17856) per the manufacturer’s instructions. These iPSC-derived CD34^+^ cells were used in subsequent qPCR and colony-forming assays.

### BFU-E and CFU-E assays.

CD34^+^ cells used in this assay were derived from iPSC-generated EBs as described above. CD34^+^ cells were plated at a density of 10^5^ cells per 35 mm dish combined with MethoCult H4034 Optimum Media (STEMCELL Technologies, 04044) and 2% FBS. Dishes were incubated at 37°C in an incubator with 5% CO_2_ and 5% O_2_ for 14 days, at which time colonies were scored for BFU-E and CFU–granulocyte, erythrocyte, monocyte, megakaryocyte (CFU-GEMM).

### KD and OE constructs for CSNK2B and lentiviral transduction.

KD lentiviral particles were purchased from Santa Cruz Biotechnology Inc., and OE construct and lentiviral particles were generated by Vector Builder. The iPSCs from CMS and non-CMS cells were transduced with polybrene (8 μg/mL, MilliporeSigma, TR-1003-G) at MOI within the range of 1 to 5 (with the titer of lentivirus ranging from 10^7^ to 10^9^). The optimal concentration was determined for the transduction and antibiotic selection by performing dose-specific kill curves. Transduced cells were selected at 0.5 μg/mL puromycin (Sigma-Aldrich, 58-58-2) or 0.5 μg/mL blasticidin (EMD Millipore, 20-335). For double KD, puromycin and blasticidin combinations were used for selection. The expression of CSNK2B in each construct was verified by qPCR at the iPSC stage as well as the iPSC-derived CD34^+^ stage.

### In vitro casein kinase inhibitor experiments.

TBB (catalog ab120988) and CX4945 (catalog S2248) were purchased from Abcam and Selleckcam, respectively. Dose-response experiments were performed with the inhibitors using the following concentrations in the colony forming assays using iPSC-derived CD34^+^ cells as described above: TBB (25 μM, 50 μM, and 100 μM) and CX4945 (2.5 μM, 5 μM, and 10 μM).

### Western blot analysis for quantification of protein levels.

Proteins were isolated using standard protein isolation protocols with RIPA buffer (Cell Signaling Technology, 9806) and protease inhibitor cocktail (Roche, 11697498001). For protein isolation, EBs at week 1 were used in this study. Through FACS analysis, we determined that at this stage, the population of erythroid cells was at the CD34^+^ stage. Antibodies against CSNK2B (Abcam, catalog ab76025), DXO (Abcam, catalog ab152135), PPP1R11 (Abcam, catalog ab171960), ZNRD1 (Santa Cruz Biotechnology Inc., catalog sc-393406), and TAP2 (Santa Cruz Biotechnology Inc., catalog sc-515576) were purchased. At the same protein concentration, GAPDH (Cell Signaling Technology, catalog 2118S) was used as the control for normalizing during quantification of the blots. In brief, 20 μg of lysate supernatant was separated by 10% sodium dodecyl sulfate-polyacrylamide gel electrophoresis and transferred to a nitrocellulose membrane. The blots were developed using enhanced chemiluminescent reagents (Bio-Rad Laboratories) and the ChemiDoc XRS+ Molecular Imager (Bio-Rad Laboratories).

### Zebrafish husbandry and maintenance.

Zebrafish (*Danio rerio*) were raised in a circulating aquarium system on a 14-hour light/10-hour dark cycle at 28.5°C, following standard husbandry procedures (67).

### Morpholino and mRNA microinjection.

The morpholino antisense oligo (MO) 5′-CGACACTTCCTCTGAGCTACTCATG-3′ was synthesized to block the translation initiation of *csnk2b*, and the 5-mismatch oligo 5′-CGAGAGTTCGTCTGACCTAGTCATG-3′ was synthesized as a specificity control (Gene Tools). For synthesizing *csnk2b* rescue mRNA that is resistant to the translation blocking MO, the full-length *csnk2b* coding sequence with 4 base pairs of silent mutations in the MO recognition region was cloned into the pCS2-vector (Azenta Life Sciences), in which the first 24 base pairs of the *csnk2b* coding sequence became 5′-ATGAGTAGCTCAGAAGAGGTCTCC-3′. The *csnk2b* capped mRNA was synthesized using the mMESSAGE mMACHINE Kit (Ambion, AM1340). Microinjection was performed on WT AB embryos at the 1- to 2-cell stages. Unless otherwise indicated, each embryo was injected with 5 ng of *csnk2b* MO and 50 pg of *csnk2b* mRNA for KD and rescue, respectively.

### Hemoglobin staining.

Embryos at 2 dpf were dechorionated and anesthetized with 0.016% tricaine (Fluka, A5040), followed by a 15-minute incubation in 0.6 mg/mL *o*-dianisidine solution (Sigma-Aldrich, D9143). This solution was prepared in 0.65% H_2_O_2_(EMD, HX0647-3), 40% ethanol (KOPTEC, 89125), and 10 mM sodium acetate (Fisher Chemical, S210-500) at room temperature. Stained embryos were washed twice with 1× PBS (Gibco, Thermo Fisher Scientific, 14200166) and then fixed in 4% paraformaldehyde (PFA) (Sigma-Aldrich, P6148). Hemoglobin signal was observed under a light microscope and quantified according to the area and intensity in the heart and common cardinal vein; embryos were categorized into normal, medium, and low hemoglobin levels.

### Data availability.

RNA-Seq data were deposited in the NCBI’s Sequence Read Archive (SRA BioProject PRJNA826881).

### Statistics.

For qPCR analysis under hypoxia and normoxia conditions, 2-tailed, unpaired *t* tests were performed. For multiple comparisons, such as for the inhibitor experiments or BFU assays, we performed 1-way ANOVA followed by Tukey’s tests. For in vivo study, χ^2^ tests were performed to examine the statistical differences of the phenotype distribution between groups. *P* < 0.05 was considered statistically significant.

### Study approval.

For human studies, each subject provided informed, written consent under IRB protocols approved by UCSD and the Universidad Peruana Cayetano Heredia, Lima, Peru. For zebrafish studies, animal protocols were approved by the UCSD Animal Research Committee to ensure compliance with all tenets of the Animal Welfare Act and Public Health Service (PHS) policy.

## Author contributions

PA, DZ, and GGH conceived the research and designed all experiments. PA performed all the experiments and contributed to writing the manuscript. DZ analyzed and interpreted the RNA-Seq data and contributed to writing the manuscript. HCT performed zebrafish experiments. FCV provided patient samples and iPSC lines as needed for the experiments. DT conceived and interpreted zebrafish experimental results. TMR conceived experiments and data interpretation related to lncRNA. DT, TMR, and GGH contributed by interpreting data and critically reviewing the manuscript. Co–first authorship and order were determined by contributions to the findings described in the manuscript.

## Supplementary Material

Supplemental data

## Figures and Tables

**Table 1:**
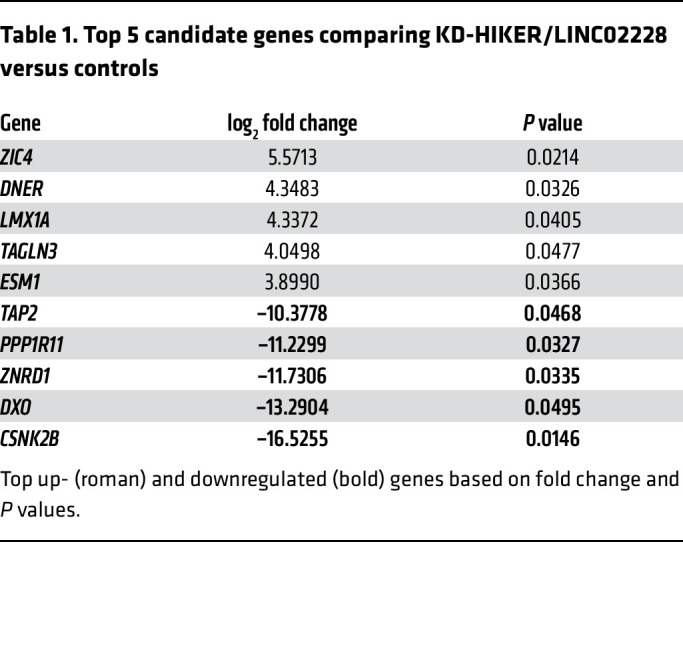
Top 5 candidate genes comparing KD-HIKER/LINC02228 versus controls

## Version 1 04/06/2023

In-Press Preview

## Version 2 06/01/2023

Electronic publication
