# Supplementary material for: Long noncoding RNA HIKER regulates erythropoiesis in Monge’s disease via CSNK2B
Source: J Clin Invest. 2023 Jun 1;133(11):e165831. doi: 10.1172/JCI165831 (PMC10231995; doi:10.1172/JCI165831)
Supplement: Supplemental data [file jci-133-165831-s014.pdf]

Supplementary information:

Supplementary Tables:

Supplementary Table 1: List of candidate lncRNAs altered by hypoxia in CMS and non-CMS cells.

Table S1. List of candidate lncRNAs altered by hypoxia treatment					
**ID**	**Symbol**	**Entrez Gene Name**	**log2 FC**	**1stRPKM**	**2ndRPKM**
**CMS**					
ENSG00000236871.6	LINC00106	long intergenic non-protein coding RNA 106	1.964	0.662	2.893
ENSG00000224328.1	MDC1-AS1	MDC1 antisense RNA 1	1.223	4.427	10.443
ENSG00000251273.1	LINC02228	long intergenic non-protein coding RNA 2228	1.009	2.203	4.544
ENSG00000277142.1	LINC00235	long intergenic non-protein coding RNA 235	-1.056	2.407	1.140
ENSG00000224259.5	LINC01133	long intergenic non-protein coding RNA 1133	-1.955	3.665	0.902
**nonCMS**					
ENSG00000260851.6	BEAN1-AS1	BEAN1 antisense RNA 1	4.954	0.000	2.173
ENSG00000226179.5	LINC00685	long intergenic non-protein coding RNA 685	4.146	0.000	2.377
ENSG00000231310.3	TBL1XR1-AS1	TBL1XR1 antisense RNA 1	2.751	0.788	5.712
ENSG00000234166.1	ARHGEF19-AS1	ARHGEF19 antisense RNA 1	2.468	0.279	2.163
ENSG00000237854.3	LINC00674	long intergenic non-protein coding RNA 674	2.101	0.584	2.773
ENSG00000267308.1	LINC01764		1.919	1.008	4.056
ENSG00000256073.3	URB1-AS1	URB1 antisense RNA 1 (head to head)	1.571	1.327	4.081
ENSG00000229851.1	ARSD-AS1	ARSD antisense RNA 1	1.530	1.652	4.905
ENSG00000236269.1	ENO1-IT1		1.488	1.153	3.373
ENSG00000249069.7	LINC01033		1.346	1.264	3.281
ENSG00000244265.1	SIAH2-AS1	SIAH2 antisense RNA 1	1.246	3.752	9.075
ENSG00000268592.3	RAET1E-AS1	RAET1E antisense RNA 1	1.146	0.912	2.074
ENSG00000204261.8	PSMB8-AS1	PSMB8 antisense RNA 1 (head to head)	1.135	3.864	8.518
ENSG00000246067.7	RAB30-AS1	RAB30 antisense RNA 1 (head to head)	1.130	1.085	2.402
ENSG00000225376.5	TMEM246-AS1	TMEM246 antisense RNA 1	1.103	1.140	2.524
ENSG00000242797.3	GLYCTK-AS1	GLYCTK antisense RNA 1	1.085	1.013	2.194
ENSG00000245148.2	ARAP1-AS2	ARAP1 antisense RNA 2	1.026	1.848	3.793
ENSG00000233661.1	SPIN4-AS1	SPIN4 antisense RNA 1	-1.067	7.193	3.344
ENSG00000279873.2	LINC01126	long intergenic non-protein coding RNA 1126	-1.199	2.234	0.948
ENSG00000264575.1	LINC00526	long intergenic non-protein coding RNA 526	-1.288	2.353	0.941
ENSG00000272414.5	FAM47E-STBD1	FAM47E-STBD1 readthrough	-1.383	5.766	2.197
ENSG00000248866.1	USP46-AS1	USP46 antisense RNA 1	-1.410	2.865	1.036
ENSG00000223534.1	HLA-DQB1-AS1	HLA-DQB1 antisense RNA 1	-1.438	4.552	1.598
ENSG00000224101.1	ELMO1-AS1	ELMO1 antisense RNA 1	-1.438	6.848	2.441
ENSG00000237883.1	DGUOK-AS1	DGUOK antisense RNA 1	-1.438	2.692	0.941
ENSG00000259521.1	INO80-AS1		-1.468	4.132	1.429
ENSG00000229372.1	SZT2-AS1	SZT2 antisense RNA 1	-1.523	2.981	0.970
ENSG00000229780.1	UBE2Q1-AS1	UBE2Q1 antisense RNA 1	-1.760	16.882	4.868
ENSG00000220925.2	IGBP1-AS2		-1.776	8.482	2.380
ENSG00000225760.1	LINC00431	long intergenic non-protein coding RNA 431	-1.924	3.673	0.893
ENSG00000237080.2	EHMT2-AS1		-1.932	6.079	1.455
ENSG00000260924.2	LINC01311	long intergenic non-protein coding RNA 1311	-2.491	2.333	0.376
ENSG00000226352.2	PSPC1-AS2		-2.608	4.238	0.541
ENSG00000239801.1	DENND6A-AS1		-2.626	6.735	0.932
ENSG00000249310.2	APOBEC3B-AS1	APOBEC3B antisense RNA 1	-3.497	2.129	0.098
ENSG00000269386.5	RAB11B-AS1	RAB11B antisense RNA 1	-4.345	2.333	0.059

Supplementary Table 2. List of specific LINC02228 downstream target transcripts.

Gene_Symbol	baseMean	log2FoldChange	lfcSE	stat	pvalue
ZIC4	99.632	5.571	2.422	2.300	0.021
DNER	116.940	4.348	2.035	2.137	0.033
LMX1A	87.065	4.337	2.117	2.048	0.041
CDH18	79.390	4.197	2.107	1.992	0.046
TAGLN3	84.095	4.050	2.045	1.980	0.048
ESM1	141.557	3.899	1.865	2.091	0.037
SLC16A14	117.895	3.875	1.902	2.038	0.042
MMRN1	467.196	3.860	1.663	2.321	0.020
GPM6A	137.902	3.859	1.860	2.075	0.038
CALB1	116.387	3.720	1.861	1.999	0.046
CNTN2	147.995	3.646	1.785	2.043	0.041
MAP2	497.426	3.640	1.599	2.276	0.023
MAP6	179.603	3.600	1.732	2.078	0.038
ILDR2	231.583	3.587	1.684	2.130	0.033
SLC1A2	184.213	3.421	1.677	2.040	0.041
FOXJ1	280.406	3.333	1.584	2.104	0.035
DCX	265.834	3.292	1.580	2.084	0.037
NAV3	238.152	3.208	1.572	2.040	0.041
NEFL	593.094	3.164	1.454	2.177	0.029
CTNNA2	282.317	3.104	1.517	2.046	0.041
NELL2	500.631	2.890	1.390	2.080	0.038
FAM84A	408.271	2.755	1.369	2.012	0.044
GPR98	1118.022	2.429	1.195	2.032	0.042
KRT7	2449.952	2.395	1.159	2.066	0.039
IGFBPL1	909.744	2.304	1.168	1.973	0.049
KIF1A	1030.216	2.274	1.152	1.975	0.048
PAPPA2	1998.311	2.118	1.077	1.967	0.049
EEF1A1	166227.896	-2.000	1.010	-1.981	0.048
EPHA7	4509.372	-2.053	1.040	-1.974	0.048
HMGB1	5532.229	-2.206	1.087	-2.030	0.042
HERC2P2	1705.768	-2.293	1.137	-2.017	0.044
CXCL14	1046.689	-2.294	1.157	-1.983	0.047
RSPO2	1699.377	-2.309	1.142	-2.022	0.043
GOLGA8A	970.402	-2.388	1.190	-2.006	0.045
FIBIN	1003.742	-2.418	1.197	-2.020	0.043
EGFLAM	2963.314	-2.450	1.172	-2.091	0.037
RPL39	7599.851	-2.470	1.165	-2.120	0.034
PKD1P1	1662.080	-2.587	1.228	-2.106	0.035
DCN	4747.156	-2.608	1.212	-2.153	0.031
RPS3A	17051.926	-2.617	1.205	-2.172	0.030

FAM45B	1154.329	-2.639	1.258	-2.097	0.036
RNU12	3100.256	-2.641	1.228	-2.150	0.032
HGF	678.513	-2.718	1.313	-2.070	0.038
FABP5	634.996	-2.734	1.323	-2.067	0.039
PDZRN4	541.804	-2.757	1.342	-2.055	0.040
SNORD116-17	705.144	-2.795	1.333	-2.097	0.036
SNORD116-19	705.144	-2.795	1.333	-2.097	0.036
GATSL2	977.185	-2.799	1.314	-2.130	0.033
PI4KAP2	646.437	-2.823	1.342	-2.104	0.035
NOMO1	3561.968	-2.832	1.281	-2.210	0.027
SERPINA1	546.770	-2.834	1.364	-2.078	0.038
SDHAP1	470.868	-2.860	1.385	-2.065	0.039
ALOX15	927.548	-2.872	1.338	-2.146	0.032
TYRP1	951.996	-2.878	1.338	-2.150	0.032
LOC440434	501.107	-2.900	1.391	-2.085	0.037
PROK1	411.111	-2.924	1.417	-2.063	0.039
BCRP3	216.022	-2.968	1.514	-1.961	0.050
PI4KAP1	439.110	-3.056	1.449	-2.109	0.035
ZNF286B	618.774	-3.099	1.430	-2.167	0.030
HLA-B	777.458	-3.116	1.418	-2.197	0.028
ODAM	283.023	-3.118	1.518	-2.054	0.040
COL21A1	845.460	-3.165	1.427	-2.219	0.027
HLA-DPB1	490.874	-3.174	1.471	-2.157	0.031
PDIA3P1	876.702	-3.189	1.431	-2.228	0.026
LOC100288162	607.773	-3.232	1.469	-2.200	0.028
MIR6723	388.199	-3.235	1.512	-2.139	0.032
ARHGEF5	570.506	-3.277	1.486	-2.205	0.027
GCSH	763.804	-3.305	1.472	-2.246	0.025
LOC440300	557.227	-3.376	1.515	-2.228	0.026
LOC100288778	474.025	-3.566	1.581	-2.255	0.024
APOC1P1	106.132	-3.836	1.902	-2.017	0.044
PLEKHM1	404.598	-3.846	1.671	-2.301	0.021
NPIPA1	348.955	-3.848	1.689	-2.278	0.023
ANXA2P1	86.201	-3.880	1.968	-1.972	0.049
ARHGEF34P	354.652	-4.002	1.726	-2.318	0.020
LOC643387	123.552	-4.152	1.950	-2.129	0.033
AHSG	287.192	-4.259	1.819	-2.342	0.019
FAM72A	156.115	-4.274	1.930	-2.214	0.027
RPL23P8	352.000	-4.317	1.805	-2.392	0.017
ROCK1P1	185.258	-4.331	1.910	-2.267	0.023
MTRNR2L2	138.920	-4.417	1.992	-2.218	0.027
ATP8B5P	73.747	-4.421	2.162	-2.045	0.041
GTF2IRD2	267.167	-4.497	1.888	-2.382	0.017

NACAP1	65.492	-4.621	2.255	-2.049	0.040
ARHGAP27	157.708	-4.699	2.034	-2.310	0.021
LOC642236	121.359	-4.737	2.105	-2.251	0.024
CRSP8P	45.242	-4.904	2.476	-1.980	0.048
PMS2L2	56.552	-4.904	2.386	-2.056	0.040
HLA-DPA1	242.897	-5.252	2.079	-2.526	0.012
GOLGA8N	44.876	-5.319	2.601	-2.045	0.041
ANKRD20A4	35.618	-5.577	2.797	-1.994	0.046
FAM35DP	215.075	-5.586	2.177	-2.566	0.010
RAB6C	184.930	-5.632	2.219	-2.539	0.011
GOLGA6L6	55.821	-5.641	2.598	-2.171	0.030
NPIPB5	170.199	-5.665	2.245	-2.523	0.012
CXADRP2	47.247	-5.992	2.781	-2.154	0.031
ZNF812	28.412	-6.262	3.183	-1.967	0.049
LOC100132057	29.096	-6.297	3.181	-1.980	0.048
HLA-DOA	175.259	-6.302	2.384	-2.644	0.008
CTAGE4	141.010	-6.577	2.509	-2.622	0.009
ZNRD1-AS1	151.271	-6.679	2.512	-2.659	0.008
LOC613037	129.699	-6.874	2.609	-2.634	0.008
POM121L1P	43.461	-6.882	3.139	-2.192	0.028
PRR21	50.986	-7.114	3.126	-2.275	0.023
DDX11L10	51.670	-7.133	3.125	-2.282	0.022
CFC1B	115.653	-7.297	2.766	-2.638	0.008
DDX11L9	58.510	-7.314	3.117	-2.346	0.019
LINC01001	117.705	-7.322	2.767	-2.647	0.008
TSPY3	64.667	-7.459	3.111	-2.397	0.017
TNXA	65.351	-7.474	3.111	-2.403	0.016
CST4	66.719	-7.504	3.109	-2.413	0.016
TSPY4	86.557	-7.882	3.097	-2.545	0.011
LOC100190986	89.293	-7.927	3.096	-2.560	0.010
MICB	102.974	-8.133	3.092	-2.630	0.009
FAM157B	22.574	-8.391	4.231	-1.983	0.047
RNU1-13P	25.310	-8.556	4.188	-2.043	0.041
REREP3	32.151	-8.901	4.107	-2.167	0.030
MOG	43.780	-9.347	4.021	-2.325	0.020
PRRT1	43.780	-9.347	4.021	-2.325	0.020
TCEB3CL	43.780	-9.347	4.021	-2.325	0.020
TAP2	486.731	-10.378	5.221	-1.988	0.047
PPP1R11	1756.707	-11.230	5.259	-2.135	0.033
ZNRD1	1242.615	-11.731	5.518	-2.126	0.034
DXO	673.797	-13.290	6.767	-1.964	0.050
CSNK2B	6345.321	-16.526	6.766	-2.442	0.015

Supplementary Table 3: List of Oligonucleotides and primers for qPCR used in the study.

	**Oligonucleotides and Primers**	
**SNO.**	**Name**	**Sequence (5' --> 3')**
1	LINC02228-ASO1	G*G*T*T*G*C*T*T*T*C*A*G*G*C*C*C*C*T*T*A
2	LINC02228-ASO2	C*T*T*C*A*C*C*A*T*G*C*T*T*G*T*G*C*T*C*T
3	LINC02228-ASO3	G*C*C*T*T*C*C*G*G*G*C*C*G*T*C*C*A*C*G*C
4	LINC00431-ASO1	T*T*T*C*C*T*C*G*G*T*C*C*A*T*G*C*A*C*A*C
5	LINC00431-ASO2	A*C*G*A*T*A*C*C*C*T*T*G*C*T*C*C*C*A*A*C
6	LINC00431-ASO3	A*C*C*G*G*C*T*C*C*C*A*T*G*G*T*G*T*C*T*T
7	LINC01133-L	GGAGCGAGATCCCTCCAAAAT
8	LINC01133-R	GGCTGTTGTCATACTTCTCATGG
9	GAPDH-L	CCACATCGCTCAGACACCAT
10	GAPDH-R	ACCAGGCGCCCAATACG
11	MDC1-AS-L	TCCCAGATGTGCCAAAGTCAG
12	MDC1-AS-R	AGCAACCCCAGTTGTCATTC
13	RAB11B-AS1-L	GGAACATGTTTACATGGACTTTGT
14	RAB11B-AS1-R	TCTTTGTTCTTGTTTGTTTTCTTTCT
15	LINC02228-L	CTGAAAGCAACCTCCAGTCC
16	LINC02228-R	GCCAGTTGCATATTGCTTCA
17	APOBEC3B-AS1-L	CTCTTACTGCTGGGCCTGTC
18	APOBEC3B-AS1-R	AGACCGTTCACCACCATCTC
19	LINC00674-L	CATTGCTGAAGTTGGACTCG
20	LINC00674-R	TCTCGCAGGCTAAACTGACC
21	SIAH2-AS1-L	GTTTCCTCCCTCCTCAATCC
22	SIAH2-AS1-R	CCTCTGCAGACGTGTATTCG

23	SZT2-AS1-L	GACCGTCATGGATAGGAAGC
24	SZT2-AS1-R	CCGAAAGAGCTGGGATAGG
25	UBE2Q1-AS1-L	CTAGAATGCAGCCTCCAACC
26	UBE2Q1-AS1-R	TAATGGGCTGGTAGCAAAGC
27	BEAN1-AS1-F	CCTGTGAGCTCTTCCTGACC
28	BEAN1-AS1-R	ACACAGGAGGCATCAAGGAG
29	TBL1XR1-AS1-F	AGCCTGACTGCTGAGTTGGT
30	TBL1XR1-AS1-R	GGAGACCCATGAAAGATGGA
31	ARHGEF19-AS1-F	CAGGGGAGGTGGTAGACAAG
32	ARHGEF19-AS1-R	ATGGGGACCATAACAGGTGA
33	CSNK2B-L	GCAATTCAAGAGCCCAGTC
34	CSNK2B-R	CCACACGATAACGACTCCT
35	DXO-L	GCAGCAAACCTGGAAGCTC
36	DXO-R	TACCCCCCTGAGAAGAGCA
37	ZNRD1-L	CATGACCTCGCCAATACTT
38	ZNRD1-R	TGAACCACAGCGAATACAG
39	PPP1R11-L	CAGCACAGAAAAAGGGAAG
40	PPP1R11-R	TTGTTCAGTGACGGTCTCG
41	TAP2-L	AGGAGCTGCTTCACCTACA
42	TAP2-R	TGAGTCAGCTCCCCTGTCT

Supplementary Figures:

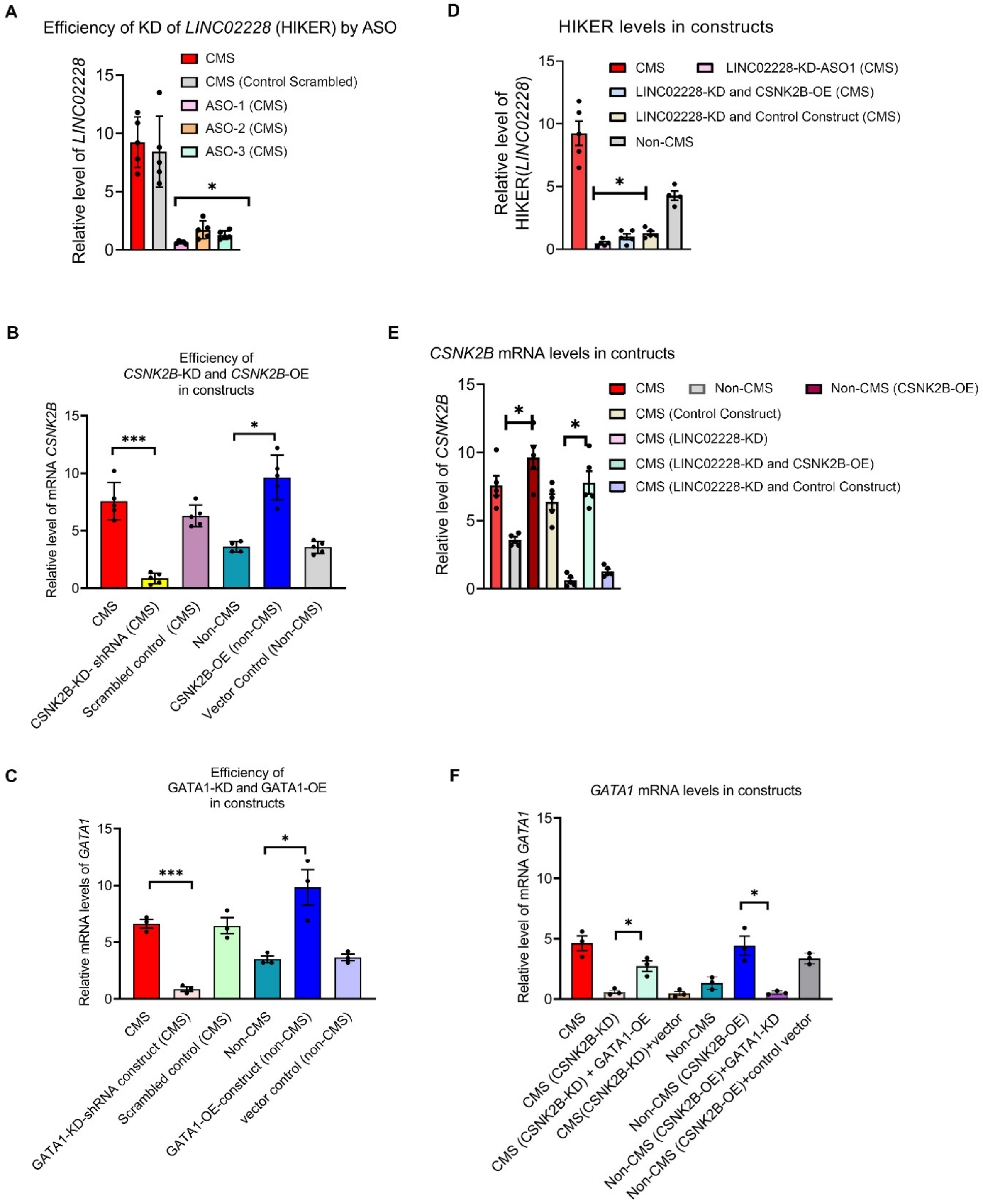

Supplementary Figure 1. KD efficiency of HIKER, CSNK2B and GATA1 determined by qPCR. The figure panels on the left (A, B, C) show the efficiency of KD or OE. FOR ASO the figure shows the KD efficiency by 3 ASO tested for KD (>80%). ASO-1 was used in subsequent experiments. The right panel figures (D, E and F) show the expression of each construct used in colony forming assays. n=3 for at least each group. ***p* value <0.001. (one way ANOVA was performed followed by Tukey tests).

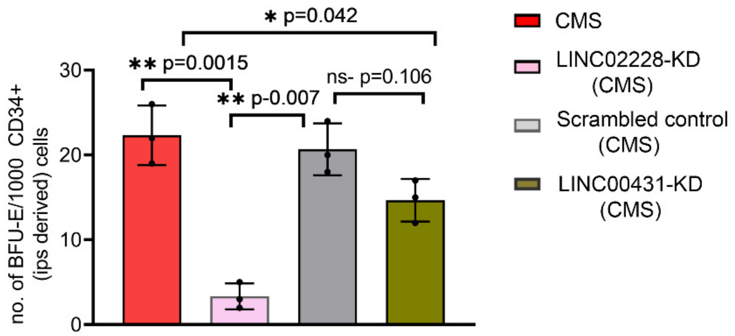

Supplementary Figure 2: Functional analysis as BFU assay of LINC02228 and LINC00431.

The graph shows BFU colonies for each group along with the controls. *p value <0.05, **p value <0.01. N=3 for each group. (one way ANOVA was performed followed by Tukey tests).

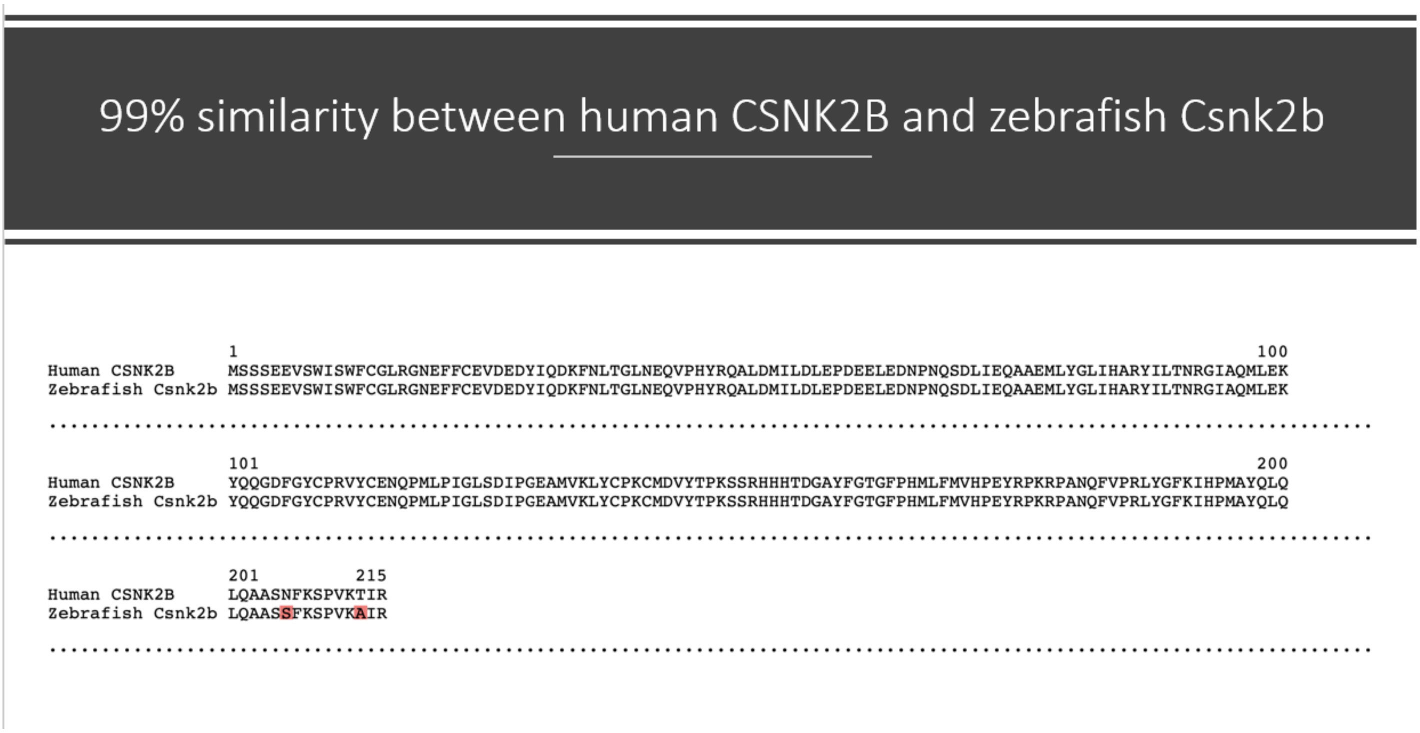

Supplementary Figure 3: Protein sequence similarity between human and zebrafish CSNK2B.

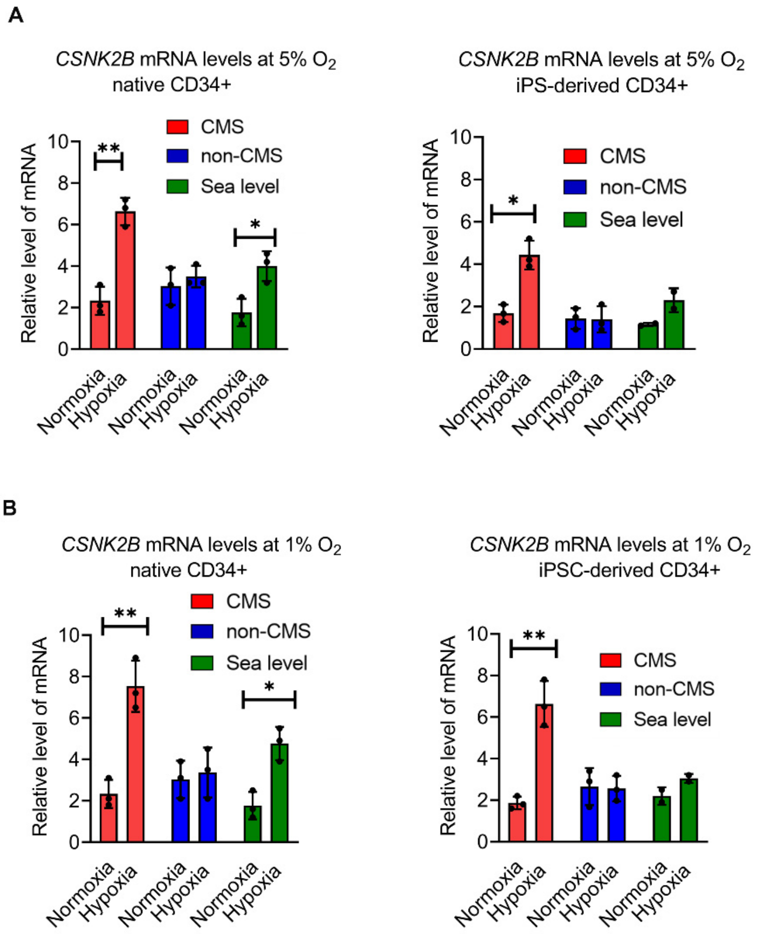

Supplementary Figure 4: CSNK2B expression levels (q-PCR) in iPSC-derived and PBMCs derived native CD34+ at 5 and 1% O_2.

**p* value <0.05, ***p* value <0.01. N=3 for each group. T-tests were performed to compare hypoxia and normoxia for each group.
